# Physical activity moderates the association between school start time and sleep duration in a cross-sectional national sample of adolescents

**DOI:** 10.1186/s44167-024-00050-y

**Published:** 2024-05-06

**Authors:** Zachary S. Farley, Mandilyn Ward, Nicole R. Giuliani, Elizabeth L. Budd

**Affiliations:** 1 Department of Counseling Psychology and Human Services, College of Education, University of Oregon, Eugene, OR, USA; 2 Prevention Science Institute, University of Oregon, Eugene, OR, USA; 3 Department of Special Education and Clinical Sciences, College of Education, University of Oregon, Eugene, OR, USA

**Keywords:** Adolescence, Sleep, School policy, Activity, Health promotion

## Abstract

**Background:**

Adolescent insufficient sleep is an endemic issue that may result in compromised functioning throughout the course of the day and is associated with increased risk for a variety of adverse outcomes. Early school start time (SST) has been consistently found to be detrimental to adolescents’ sleep achievement on school nights. However, there are logistical barriers to changing SST. Evidence supports daily engagement in moderate-to-vigorous intensity physical activity (MVPA) to enhance adolescents’ nightly sleep achievement. However, the role of MVPA in the association between SST and sleep duration is unknown. This study examines the potential moderating effect of MVPA in the association between SST and sleep duration on a typical school day among adolescents.

**Methods:**

This study examined data (collected in April and October 2014) from a national sample of 1132 adolescents (*m*_*age*_ = 14.5 years) living in the United States from the Family Life, Activity, Sun, Health, and Eating study, a cross-sectional, internet-based survey. First, three linear regressions were computed to examine bivariate associations between SST, MVPA, and sleep duration while controlling for participant sex, race and ethnicity, household income, school level, and the presence of a TV in the bedroom. Next, a three-step multiple regression was computed with sleep duration as the dependent variable, and the final step included an interaction term between SST and MVPA.

**Results:**

Later SST (*b*_1_ = 0.41, *p* < 0.001) and increased MVPA (*b*_1_ = 0.39, *p* < 0.001) were both associated with increased sleep duration, while SST and MVPA were not significantly associated. In the final multiple regression model, which included the interaction term, school day MVPA moderated the positive association between SST and school night sleep duration (*b*_1_ = − 3.7, *p* < 0.05), such that the greater the MVPA on a typical school day, the weaker the positive association between early SST and sleep duration. In post-hoc analysis, the interaction effect was only significant for females and not males.

**Conclusions:**

The significant buffering effect of MVPA on the association between SST and sleep duration suggests that in the absence of SST changes, promoting MVPA among adolescents may be a promising strategy to mitigate insufficient sleep among US adolescents.

## Background

The American Academy of Sleep Medicine recommends adolescents achieve at least 8 h of sleep per night for optimal health [1]. However, less than 25% of adolescents in the United States (US) regularly achieve the minimum nightly sleep recommendation [2]. These unfavorable rates contributed to the American Academy of Pediatrics’ Adolescent Sleep Working Group in declaring adolescents’ insufficient sleep as an endemic issue in need of more research to inform mitigation strategies [3]. Insufficient sleep among adolescents may result in compromised physical and cognitive functioning throughout the course of the day and is associated with increased risk for depressive symptoms, behavioral issues, poor academic performance, accidental injury and/or death, and, over time, the development of chronic diseases [3].

Adolescents undergo biological changes associated with the onset of puberty, a critical period of development for a series of transformations affecting behavior, cognition, and emotion regulation, among others [4]; one of these changes is a shift in sleep–wake cycles. This shift in sleep–wake cycles results from changes to the sleep homeostatic and circadian timing systems and is denoted by a delay of up to two-hours from cycles experienced earlier in childhood [5]. These changes can result in a biological propensity for adolescents to become tired and fall asleep later in the night [5]. Thus, there may be a need for additional time in the morning for adolescents to achieve the recommended minimum of 8-h of nightly sleep.

Each year, there are upwards of 27-million students enrolled in US public secondary schools (i.e., grades 6–12) [6]. Through the lens of the Whole School, Whole Community, Whole Child model (WSCC) [7], the school-setting—a physical environment influenced by external community and social-political factors (e.g., district, state, or federal policy)—provides an opportunity to promote adolescents’ health through data-informed decision making and policy changes. During the school week, school start time (SST) policies directly influence the time available for adolescents to achieve sufficient sleep by dictating morning wake times. Extant literature has consistently found that earlier SST is associated with lesser nightly sleep among adolescents, and that delaying SSTs to accommodate the sleep–wake shift experienced by adolescents could be effective in increasing their nightly sleep achievement [8–10]. In fact, addressing early SST is now recognized in *Healthy People 2030* through the inclusion of a new objective, to “Increase the proportion of secondary schools with a start time of 8:30 a.m. or later” [11]. Despite the evidence and recommendations supporting later SSTs, most secondary schools in the US—83% of middle schools and 93% of high schools—have not adopted this SST recommendation [12]. The California Teachers Association and the California Schools Board Association recently cited barriers preventing the uptake of later SSTs when the state of California presented—and later passed (2019) and enacted (Fall of 2023) —legislation (SB-328 Pupil Attendance: School Start Time) requiring middle and high schools in the state of California to start no earlier than 8:00 and 8:30 a.m., respectively [13]. Example barriers included the need to accommodate childcare needs resulting from disruptions in scheduling for working parents—including equity concerns for challenges faced by low-income families with inflexible working hours—as well as extracurricular activities going later into the evening [14]. Given persistent adherence to early SST and barriers to implementation of delayed SST, there is a need to examine other modifiable factors that could alleviate the insufficient sleep endemic facing US adolescents.

Engaging regularly in moderate-to-vigorous intensity physical activity (MVPA) has been shown to be significantly associated with improved health and well-being among adolescents [15–17]. Evidence also supports that an increase in daytime MVPA is associated with earlier sleep onset, improved sleep quality, and longer nightly sleep duration [17–19], among other beneficial sleep outcomes. However, there is a gap in empirical understanding regarding how MVPA interacts with SST in association with adolescents’ sleep duration on school nights. Thus, in response to the American Academy of Pediatrics’ call for more research to inform mitigation strategies [3], the current study aims to develop a greater understanding of the dynamic interplay between SST, sleep duration, and MVPA to address the insufficient sleep endemic facing US adolescents. To the authors’ knowledge, this study is the first to examine the interaction between SST and MVPA as they relate to adolescents’ sleep duration on a typical school day.

Using data from a national sample of US adolescents in the Family Life, Activity, Sun, Health, and Eating (FLASHE) study [20], the following research questions were addressed:

(1) What are the associations between SST, sleep duration, and MVPA among adolescents on a typical school day? and (2) Does MVPA moderate the association between SST and sleep duration among adolescents on a typical school day? Controlling for participant sex [21–23], race and ethnicity [24], household income [22], school level [25], and presence of a TV in the bedroom [26], we hypothesize that, as SST is later, sleep duration and MVPA will increase [9, 10], and as MVPA increases so will sleep duration [17–19]. Controlling for the same covariates, we hypothesize the hypothesized positive association between SST and sleep duration will be significantly moderated by MVPA.

## Methods

### Data source

This study uses data from the FLASHE study, and the datasets and materials supporting the conclusions of this article are available on National Cancer Institute’s website (http://cancercontrol.cancer.gov/brp/hbrb/flashe.html) [20]. FLASHE is a cross-sectional internet-based survey administered by Westat, Inc., funded by the National Cancer Institute. FLASHE examines cancer-related health behaviors in a national sample of US parent-adolescent dyads. FLASHE data collection occurred between April and October 2014. Participants were recruited through Ipsos Consumer Opinion Panels, and sample selection employed balancing techniques to provide a national US sample, but not a nationally representative sample. A full description of recruitment and informed consent [27] and other study methods [28] have been previously published, and additional information can be found on National Cancer Institute’s, FLASHE webpage (https://cancercontrol.cancer.gov/brp/hbrb/flashe-study) [20]. The current study includes responses predominately from adolescents (ages 12–17.5 years; adolescent demographic survey data and adolescent physical activity survey data) who were living at least 50% of the time with a parent/guardian who is a member of Ipsos Consumer Opinion Panels; only household income derived from the parent demographic survey data [20]. FLASHE study and protocol were reviewed and approved by the US Office of Management and Budget, NCI’s Special Studies Institutional Review Board (IRB), and Westat’s internal IRB.

### Measures

FLASHE employed measures originally used by Lytle to obtain time in bed on weekdays during the school year, a proxy for usual school night sleep duration [29], which has also been used as a proxy for nightly sleep duration by others examining FLASHE data. Adolescents were asked to report: [On weekdays during the school year] *“What time do you usually go to bed in the evening?”;* and *“What time do you usually get out of bed in the morning?”* Sleep duration for a typical school night was calculated as the amount of time in minutes elapsed from time in bed to time out of bed for Sunday through Thursday nights.

SST was obtained by adolescents responding to the question, *“What time does your school day typically start?*”. In the current study SST was calculated into minutes after 12:00 AM (i.e., midnight).

MVPA was measured using the Youth Activity Profile (YAP) [30], which has been validated for use in large-sample studies [31]. Specifically, the YAP estimates adolescents’ daily minutes of in-school and out-of-school MVPA for a typical weekday, and MVPA on the weekends, by asking participants to report about activities in each of those domains during the past seven days. The YAP questionnaire uses, “things that involve a lot of walking, running or moving around” as the definition of physical activities, while also including examples like biking, playing a sport, dancing, and outside play (see supplementary material of the cited article for questions and methods used to estimate domain specific MVPA estimates) [30]. In the current study, we were specifically interested in total minutes of MVPA for a typical weekday during the school year (i.e., typical school day), which was obtained from summing the generated estimates from weekday out-of-school and weekday in-school MVPA for a typical weekday. The self-reported YAP responses and resulting estimates for FLASHE in-school and out-of-school MVPA data were validated with accelerometer-obtained estimates in a small subsample of FLASHE participants (n = 118) and were found to be statistically equivalent using the 10–15% equivalence zone criteria, despite being marginally overestimated [31].

Study analyses included covariates that have been associated with sleep, SST, and/or MVPA among adolescents, including sex assigned at birth [21–23], race and ethnicity [24], household income [22], school level [25], and presence of a TV in the bedroom [26]. Adolescents self-reported their sex assigned at birth (male or female), school level (middle or high school), race, ethnicity, and the presence of a TV in the bedroom (yes or no). Race and ethnicity were combined by FLASHE into a single, combined race and ethnicity variable (e.g., Hispanic, non-Hispanic Black, non-Hispanic White, non-Hispanic Other). In the current study this combined race and ethnicity variable was dichotomized into monoracial non-Hispanic White (n = 740, 65.4%) and all other races and ethnicities (n = 392, 34.6%) for analyses for two reasons: (1) due to small subgroup sample sizes for non-White racial and ethnic groups (10% Hispanic, n = 110; 16% non-Hispanic Black, n = 180; and 9% non-Hispanic Other, n = 102) in which influential cases would have greater influence on findings than potentially influential cases when levels are collapsed to represent all other races and ethnicities and, (2) despite evidence from the literature suggesting differences in the sleep duration of school-aged children by race and ethnicity, main findings point to the difference between White youth and minority youth [24]. Parents reported their annual household income, which was dichotomized as < $100,000 and ≥ $100,000 in FLASHE data.

### Preliminary sample

Merging of the adolescent and parent datasets by participant ID resulted in a total of 1649 cases. Cases reporting SST before 6:00 a.m. and after 10:00 a.m. were eliminated (*n* = 165; 10%), as they were approximately two hours outside the average SST for secondary schools in the US (8:08 a.m.) [12]. Cases missing any responses for variables used in analyses were excluded in the following order: sex (*n* = 33), race and ethnicity (*n* = 15), household income (*n* = 21), presence of TV in the bedroom (*n* = 192), MVPA (*n* = 26), and sleep duration (*n* = 23). This decision to remove cases with missing responses was made to maintain consistency with prior publications having used FLASHE data, so that findings of the current study can be taken in context. Removal of cases with missing observations resulted in a preliminary sample of 1174 adolescent respondents (See sample flowchart diagram in Additional file 1).

### Preliminary analysis

The initial sample was used to conduct a preliminary multiple regression analysis with sleep duration as the dependent variable, grand-mean centered SST (cSST) and grand-mean centered MVPA (cMVPA) and an interaction term between cSST and cMVPA (cSST*cMVPA) as independent variables, while also controlling for study covariates, to identify and eliminate multivariate outliers. Although age was originally considered as a covariate, it was eliminated prior to this preliminary analysis due to multicollinearity issues with school level and MVPA. After this preliminary analysis, multivariate outliers were identified and removed if they violated Cook’s distances criterion of 4/*N* [32]. Following the removal of multivariate outliers, the resulting final sample was used to investigate the study’s research questions. SST and MVPA were re-centered following the removal of outliers.

### Research question 1 analysis plan

To test the hypothesis for the first research question, unadjusted zero-order correlations between SST, sleep duration, and MVPA were computed. Next, three linear regressions were conducted to assess bivariate associations between SST, sleep duration, and MVPA while controlling for participant’s sex, race and ethnicity, household income, school level, and presence of a TV in the bedroom.

### Research question 2 analysis plan

To test the hypothesis for the second research question, a three-step multiple regression was conducted with sleep duration as the dependent variable and participant’s sex, combined race and ethnicity, household income, school level, and presence of a TV in the bedroom as covariates. In step one, only covariates were included, followed by step two, where raw SST and raw MVPA were added as independent variables. Finally, an interaction term between cSST and cMVPA was added. All assumptions of multiple regression were assessed. All statistical analyses were conducted in R-studio version 3.5.1 [33].

### Post-hoc analysis plan

To address a pertinent inquiry raised during review, we conducted a post-hoc analysis to delve deeper into the examination of the association between SST and sleep with MVPA as a moderator by looking at differences in findings between male and female participants, separately. To do so, we subset the data by sex (e.g., males and females) and ran the full model that included sleep duration as the dependent variable, the interaction term between cSST and cMVPA, and controlled for participant-reported combined race and ethnicity, school level and the presence of a TV in the bedroom, and parent-reported household income. Specifically, we were interested in assessing the differences in effects for the interaction term between male and female participants.

## Results

### Sample

Using Cook’s distances criterion to identify multivariate outliers from the preliminary multiple regression analysis resulted in the removal of 42 cases. The final sample included 1132 adolescent respondents. Excluded participants were more likely to be male, in middle school, report longer sleep duration, and report a later SST than included participants (*ps* < 0.05).

Table 1 presents characteristics of the final sample. Most participants in the sample identified as female (52%), non-Hispanic White (65.4%), in high school (60.9%), and reported the presence of a TV in their bedroom (55.4%). The final sample had an average: age of 14.5 years old (*sd* = 1.6); nightly sleep duration of 510.42 min (*sd* = 61.8); SST of 7:57 AM (*sd* = 32.2 min); and approximately 112.56 (*sd* = 22.8) minutes of MVPA for a typical school day.

### Research question 1 results

In the linear regression models examining bivariate associations between key study variables while controlling for covariates, every 1-min increase in SST was associated with an additional 0.41 min (~ 24.6 s) of sleep duration, *b*_1_ = 0.41, *SE*(*b*_1_) = 0.053, *t* (1125) = 7.72, *p* < 0.001, sr^2^ = 0.04, with the model accounting for 17.0% of the variance sleep duration, R^2^ = 0.170, *F*(6, 1125) = 38.51, p < 0.001; and every 1-min increase in MVPA was associated with an additional 0.39 min (~ 23.4 s) of sleep duration, *b*_1_ = 0.39, *SE*(*b*_1_ = 0.098, *t* (1125) = 3.98, *p* < 0.001, sr^2^ = 0.01, with the model accounting for 13.9% of the variance in sleep duration, R^2^ = 0.139, *F*(6, 1125) = 30.15, p < 0.001. MVPA was not significantly associated with SST (*p* > 0.05).

### Research question 2 results

**Fig. 1:**
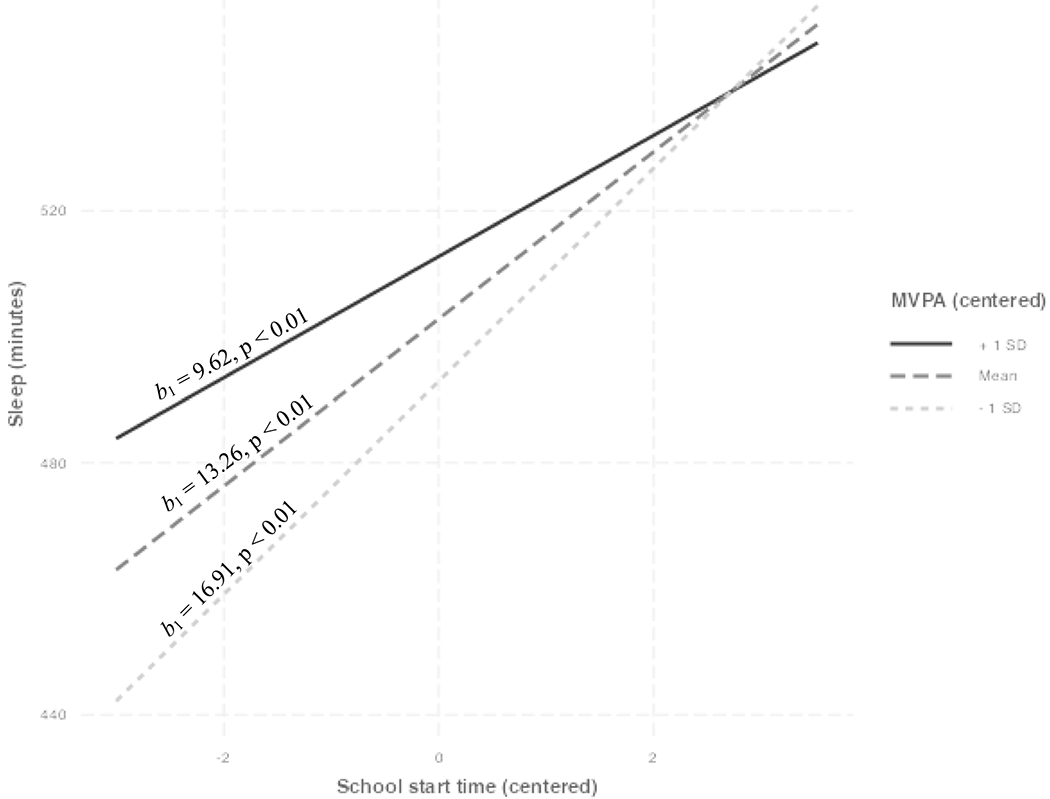
Simple slopes depicting MVPA as moderator in the association between SST and sleep

Results of the three-step multiple regression can be seen in Table 2. The final model, which included the interaction term between cSST and cMVPA, accounted for approximately 18.8% of the variance in sleep duration, *R*^2^ = 0.188 *F*(8, 1123) = 32.44, *p* < 0.001. The interaction term was negatively associated with sleep duration (p < 0.05), indicating a moderating effect of MVPA on the association between SST and sleep duration, *b*_1_ = − 3.65, *SE*(*b*_1_) = 1.74, *t* (1123) = − 2.09, *p* < 0.01, sr^2^ = < 0.01. Figure 1: Simple slopes show that as MVPA increases, the strength of the association between SST and sleep duration weakens: −1SD MVPA, *b*_1_ = 16.91, *SE*(*b*_1_) = 2.35, *t* (1123) = 7.19, *p* < 0.01.; mean MVPA, *b*_1_ = 13.26, *SE*(*b*_1_) = 1.69, *t* (1123) = 7.83, *p* < 0.01; + 1SD MVPA, *b*_1_ = 9.62, *SE*(*b*_1_) = 2.50, *t* (1123) = 3.85, *p* < 0.01.

### Post-hoc analysis results

Results of the post-hoc analysis to examine potential sex differences in the association between SST and sleep duration, while including the interaction term between cSST and cMVPA show that there are differences in the effect of the interaction term by participant sex (see Table 3 for post-hoc analyses results). For females in the sample, the model accounted for 16.4% of the variance in sleep duration, *R*^2^ = 0.164 *F*(7, 580) = 16.29, *p* < 0.001, and the interaction term was negatively associated with sleep duration, *b*_1_ = − 6.12, *SE*(*b*_1_) = 2.62, *t* (580) = − 2.33, *p* < 0.05, sr^2^ = 0.01, such that as MVPA increases the association SST and sleep weakens. For males in the sample, the model accounted for 22.3% of the variance in sleep duration, and, in contrast to the female subgroup, the interaction term was not associated with sleep duration, suggesting that MVPA does not moderate the association between SST and sleep duration for the subset of male participants.

## Discussion

Addressing the insufficient sleep endemic among US adolescents is important for improving population health [3]. Findings from this study provide novel insights into the interplay among SST, sleep duration, and MVPA among adolescents that can inform public health strategies to promote sleep and, in turn, better health outcomes for adolescents.

Consistent with our hypothesis and prior research, the results of this study indicate earlier SSTs are associated with reduced school night sleep duration [8–10]. Aligning with recommendations from *Healthy People 2030* [11] and findings from extant literature [8–10], the findings of the current study support the implementation of later SSTs to promote adolescent sleep achievement. Although the current study did not assess where the increase in sleep duration was obtained because of later SSTs (i.e., earlier sleep onset vs. later wake time), prior investigations attribute increased sleep duration to the availability of additional time in the morning to attain sleep [8–10], and future examinations using longitudinal data should assess where the increase in sleep duration is obtained. Despite the empirical support for later SSTs for secondary schools, the many barriers and concerns presented by teachers, school and district administrators, and parents highlight the difficulty of delaying SSTs in practice [14]. Research is needed on the school and community supports necessary for effective implementation of delayed SST and whether the benefits truly outweigh the costs.

The next several years, following California’s delayed SST legislation [13], California’s district and school administrators can use the WSCC [7] to better evaluate, address, and strategize how to best address structural and logistical challenges to implementing delayed SST across the state. For example, these professionals could target constructs of the WSCC model by garnering community involvement and family engagement to ascertain how changes to SST affected families and workplaces. This would enable greater understanding for how other schools and school districts can better address challenges faced by students, families, and the communities in which they live. Ultimately, this process would aid in supporting the objective of the WSCC model, which is to coordinate policy, process, and practice to improve students’ learning, health, and wellbeing [7].

Also consistent with our hypothesis, the current study shows that increases in MVPA are associated with increases in sleep duration, which contributes to one side of a mixed evidence base. Some findings in the literature indicate increasing daytime MVPA to be associated with delayed sleep onset among adolescents [34, 35], leading to lesser nightly sleep. However, Thomas and colleagues [35] posit that these effects may be related to the time of day in which MVPA is performed and individuals’ circadian timing systems and sleep timing preferences as opposed to the net amount of MVPA performed during the day. In the current study, adolescents engaging in greater amounts of MVPA on a typical school day reported longer nightly sleep. This finding is consistent with other findings [15, 16, 18] and supports the *Physical Activity Guidelines for Americans* [17] advocacy for the promotion of physical activity as a viable strategy by which to increase nightly sleep achievement among US adolescents.

Novel to the literature, study findings support our hypothesis in that MVPA moderated the association between SST and sleep duration among adolescents on a typical school day. Specifically, as MVPA increases, the strength of association between SST and sleep duration weakens; one standard deviation increase in MVPA (*sd* = 28.83 min) was associated with approximately three minutes and twelve seconds of additionally nightly sleep when holding SST constant. Prior studies have identified associations between SST and sleep duration and their association with academic outcomes [36]; between physical education and subsequent sleep outcomes [37]; or have provided evidence from epidemiological studies pointing to regular physical activity as the most important sleep promoting behavior for the public [38]. The current study advances this literature and points to MVPA promotion as a promising strategy that may support adolescents’ sleep achievement in the absence of changes to SST, despite the historical difficulty of increasing MVPA among adolescents. Additionally, MVPA’s myriad benefits to adolescents’ health, development, and wellbeing provide further justification for schools and communities to prioritize and invest in physical education, accessible afterschool physical activity opportunities, and built environment improvements to support active transportation.

The post-hoc analysis shows that there are differences in the effect of the interaction term on sleep duration by participant sex, such that for girls the cSST*cMVPA interaction term was negatively associated with sleep duration (p < 0.05), with a sr^2^ of 0.01 indicating a small partial effect. Whereas, for male participants the interaction term was not associated with sleep duration (p > 0.05). Importantly, males and females in the sample have similar means and standard deviations for MVPA, contrary to the body of evidence showing that adolescents females, on average, engage in lesser amounts of MVPA when compared to adolescent males [15–18]. Thus, it appears as if these differences between male and female participants is a function of what is happening in the associations between these variables and is not a product of the variables themselves. Also, the variable with largest partial effects for both the female and male subsets was the cSST variable (females, sr^2^ = 0.03, a small partial effect; males, sr^2^ = 0.06, a small-to-moderate partial effect), followed by the cMVPA variable (females, sr^2^ = 0.01, a small partial effect; males, sr^2^ = 0.03, a small partial effect) reaffirming the importance of considering delaying SSTs and promoting opportunities to engage in MVPA to improve adolescent sleep outcomes. Lastly, school level (high school or middle school) presents as another important factor to consider in promoting positive sleep outcomes, where for both females (sr^2^ = 0.03) and males (sr^2^ = 0.01) in our sample, school level had a small but significant partial effect on sleep duration. This could be explained in part by the association between school level and SST but could also be explained further by participants stage of pubertal development, which could not be assessed in this study.

### Limitations

The current study has several strengths, including the use of a national sample of US adolescents and a novel examination of MVPA as a moderator in the association between SST and sleep duration. Limitations include the cross-sectional study design, which prohibits causal inferences; longitudinal studies are needed to establish temporal associations among key study variables, which would better support examining MVPA as a potential mediator in the association between SST and sleep duration. Study findings may not represent adolescents who reported as male, in middle school, with later SSTs and longer nightly sleep durations, as they were disproportionately removed from the sample for having missing responses. Additionally, all measures were self-report, which are more prone to bias and error compared with objective measurement in cross-sectional studies [39]. This may be apparent in the current study as the mean sleep duration and MVPA exceeded daily recommendations [1, 17]—self-report measures used to estimate sleep duration and MVPA are prone to overestimation—and the average SST was later than the national average from the same year as FLASHE data collection [12]. The overestimation of sleep duration in the current study is undoubtedly due to the use of time in bed as a proxy for sleep duration, which fails to capture the actual length of sleep, but has been used in other studies—using FLASHE data—as a proxy for sleep duration [40]. Extant literature suggests that sleep onset and wake time are also important components of sleep outcomes in examination of associations with health benefits, as well as sleep quality measures (e.g., restfulness, feeling the need for more sleep, sleep depth, etc.) [41]. However, the current study limited examination to sleep duration because it is the sleep variable presented in the American Academy of Sleep Medicine’s recommendations for adolescent sleep [1] and is highlighted in the American Academy of Pediatrics declaration of an insufficient sleep endemic among adolescents [3]. For MVPA, despite the likely overestimation of MVPA among this sample of adolescents, the trends are likely to be consistent, wherein adolescents who are more active report more MVPA minutes than adolescents who are less active. Furthermore, in a subsample of FLASHE participants, self-report (via YAP) and objective (via accelerometry) estimates of MVPA were found to fall within the 10–15% equivalence zone criteria [31]. It is also unlikely that respondents have identical sleep/wake-times and MVPA across the course of any given school week, but these day-to-day deviations could not be accounted for in this study. Furthermore, prior research [34, 35] indicates timing, modality, and setting characteristics of MVPA performance to be influential to observed changes in sleep outcomes, but the current study was unable to account for these components and characteristics. Objective measures for sleep and MVPA using accelerometers would strengthen the validity of findings. Lastly, FLASHE data were collected nearly 10 years ago (i.e., 2014), but evidence suggests the national averages for all three key study variables from 2014 to the most recent data are comparable (SST [12, 42]; Sleep Duration [2]; MVPA [2]).

## Conclusions

The current study offers novel findings on the interplay between SST, MVPA, and sleep duration among adolescents in the US. MVPA may be a promising strategy by which to promote school night sleep achievement among adolescents regardless of SST. Given cited barriers to delaying SST and other benefits of MVPA for adolescents’ health and well-being, MVPA promotion could serve as a potential first step and be an alternative strategy by which to mitigate insufficient sleep among US adolescents, particularly in the absence of changes to SST.

## Supplementary Material

Inclusion-Exclusion Flowchart **Additional file 1.** Inclusion-Exclusion Flow Chart for Final Sample.

## Figures and Tables

**Table 1:** Characteristics of adolescent participants (N = 1132) from the FLASHE study sample.

Variable	Mean (*SD*)	Skewness	Kurtosis	Min., Max

Age	14.5 (1.6)	−0.02	−1.18	12, 17
Sleep Duration (minutes) ^a^	510.42 (61.83)	−0.10	0.24	300, 720
SST (minutes) ^b^	07:56 (32.15)	0.28	0.63	380, 590
MVPA (minutes)	112.56 (22.83)	−0.03	−0.29	40.82, 183.17

Variable	N	%		

Sex				
Female	588	52		
Male	544	48		
Combined Race & Ethnicity ^c^				
NH-White Only ^d^	740	65.4		
NH-Black Only	180	15.9		
Hispanic	110	9.7		
NH-Other	102	9.0		
Household Income				
<$100,000	889	78.5		
$100,000+	243	21.5		
School Level				
High	689	60.9		
Middle	443	39.1		
TV in Bedroom				
Yes	630	55.4		
No	507	44.6		

Note. N = 1132; reflects the final sample used for data analysis, after data cleaning.

a Reflects sleep duration, in minutes, for a typical school night.

b In the table, SST is reported in hours:minutes but was used in minutes for analyses to reflect the scales for sleep duration and MVPA, which are reported in minutes. For reference, a SST of 07:56 is the same as 476.50 minutes.

c In the current study, combined race & ethnicity was recorded dichotomously as Non-Hispanic White only and All Other Races and Ethnicities

d NH = Non-Hispanic.

**Table 2:** Multiple regression results: MVPA as a moderator in the association between SST and sleep duration

Predictor	*b*	*b* 95% CI [LL, UL]	*sr* ^*2*^	*sr*^*2*^ 95% CI [LL, UL]	Fit
(Intercept)	489.77**	[482.70, 496.85]			
Sex ^a^	7.03*	[0.23, 13.84]	.00	[−.00, .01]	
Race/Ethnicity ^b^	−8.90*	[−16.12, −1.69]	.00	[−.00, .01]	
Household Income ^c^	−7.74	[−16.09, 0.62]	.00	[−.00, .01]	
School Level ^d^	40.07**	[33.12, 47.02]	.10	[.07, .13]	
Bedroom TV ^e^	14.18**	[7.25, 21.12]	.01	[.00, .02]	
					*R*^*2*^ = .126**
					95% CI [.09,.16]

(Intercept)	249.86**	[196.50, 303.23]			
Sex ^a^	6.60*	[0.02, 13.17]	.00	[−.00, .01]	
Race/Ethnicity ^b^	−11.12**	[−18.11, −4.13]	.01	[−.00, .02]	
Household Income ^c^	−6.20	[−14.29, 1.89]	.00	[−.00, .01]	
School Level ^d^	23.17**	[14.27, 32.08]	.02	[.00, .03]	
Bedroom TV ^e^	13.54**	[6.83, 20.25]	.01	[.00, .02]	
SST_min	0.42**	[0.32, 0.52]	.05	[.02, .07]	
MVPA_tot	0.42**	[0.24, 0.61]	.01	[.00, .03]	
					*R*^*2*^ = .185**
					95% CI [.14,.22]

(Intercept)	497.43**	[490.18, 504.68]			
Sex ^a^	6.72*	[0.15, 13.29]	.00	[−.00, .01]	
Race/Ethnicity ^b^	−10.98**	[−17.96, −3.99]	.01	[−.00, .02]	
Household Income ^c^	−6.02	[−14.10, 2.05]	.00	[−.00, .01]	
School Level ^d^	23.52**	[14.62, 32.42]	.02	[.00, .03]	
Bedroom TV ^e^	13.22**	[6.51, 19.93]	.01	[−.00, .02]	
cSST	13.26**	[9.94, 16.59]	.04	[.02, .07]	
cMVPA	9.90**	[5.60, 14.20]	.01	[.00, .03]	
cSST*cMVPA	−3.65*	[−7.06, −0.24]	.00	[−.00, .01]	
					*R*^*2*^ = .188**
					95% CI [.14,.22]

*Note. N* = 1132; reflects the final sample used for analysis, after data cleaning.

A significant *b*-weight indicates the semi-partial correlation is also significant. *b* represents unstandardized regression weights. *sr^2^* represents the semi-partial correlation squared. *LL* and *UL* indicate the lower and upper limits of a confidence interval, respectively.

* indicates p < .05.

** indicates p < .01.

a ref = female.

b ref = Non-Hispanic White Only.

c ref = <$100,000.

d ref = high school.

e ref = yes.

**Table 3:** Post-hoc regression results: Comparing females and males

Predictor	*b*	*b* 95% CI [LL, UL]	*sr* ^*2*^	*sr*^*2*^ 95% CI [LL, UL]	Fit
**Female Subset**					
(Intercept)	498.09**	[488.25, 507.92]			
Race/Ethnicity ^a^	−9.59	[−20.16, 0.97]	.00	[−.01, .01]	
Household Income ^b^	−10.71	[−22.27, 0.85]	.00	[−.01, .01]	
School Level ^c^	26.92**	[14.01, 39.83]	.02	[.00, .05]	
Bedroom TV ^d^	10.63*	[0.85, 20.42]	.01	[−.01, .02]	
cSST	11.68**	[6.71, 16.66]	.03	[.01, .06]	
cMVPA	9.32**	[2.81, 15.82]	.01	[−.00, .03]	
cSST*cMVPA	−6.12*	[−11.27, −0.96]	.01	[−.01, .02]	
					*R*^*2*^ = .164**
					95% CI [.10, .21]

**Male Subset**					
(Intercept)	504.59**	[496.11, 513.07]			
Race/Ethnicity	−12.90**	[−22.16, −3.64]	.01	[−.00, .03]	
Household Income	−0.54	[−11.91, 10.83]	.00	[−.00, .00]	
School Level	18.29**	[6.01, 30.58]	.01	[−.00, .03]	
Bedroom TV	15.64**	[6.36, 24.93]	.02	[−.00, .03]	
cSST	14.90**	[10.44, 19.36]	.06	[.03, .10]	
cMVPA	11.37**	[5.63, 17.11]	.02	[.00, .04]	
cSST*cMVPA	−1.09	[−5.65, 3.47]	.00	[−.00, .00]	
					*R*^*2*^ = .223**
					95% CI [.16, .27]

*Note*. A significant *b*-weight indicates the semi-partial correlation is also significant. *b* represents unstandardized regression weights. *sr^2^* represents the semi-partial correlation squared. *LL* and *UL* indicate the lower and upper limits of a confidence interval, respectively.

* indicates p < .05.

** indicates p < .01.

a ref = Non-Hispanic White Only.

b ref = <$100,000.

c. ref = high school.

d. ref = yes.

## Data Availability

The data that support the findings of this study are available from the National Cancer Institute, but restrictions apply to the use of these data. Data and description of restrictions are available at http://cancercontrol.cancer.gov/brp/hbrb/flashe.html.

## Abbreviations

SST: School start time
cSST: Grand-mean Centered School Start Time
MVPA: Moderate-to-vigorous Intensity Physical Activity
cMVPA: Grand-mean Centered Moderate-to-vigorous Intensity Physical Activity
cSST*cMVPA: Interaction term for cSST and cMVPA
WSCC: Whole School, Whole Community, Whole Child
US: United States
FLASHE: Family Life, Activity, Sun, Health, and Eating
IRB: Institutional Review Board
YAP: Youth Activity Profile
