# Supplementary figures and images for: Physical activity moderates the association between school start time and sleep duration in a cross-sectional national sample of adolescents
Source: J Act Sedentary Sleep Behav. 2024 May 6;3:11. doi: 10.1186/s44167-024-00050-y (PMC11259015; doi:10.1186/s44167-024-00050-y)

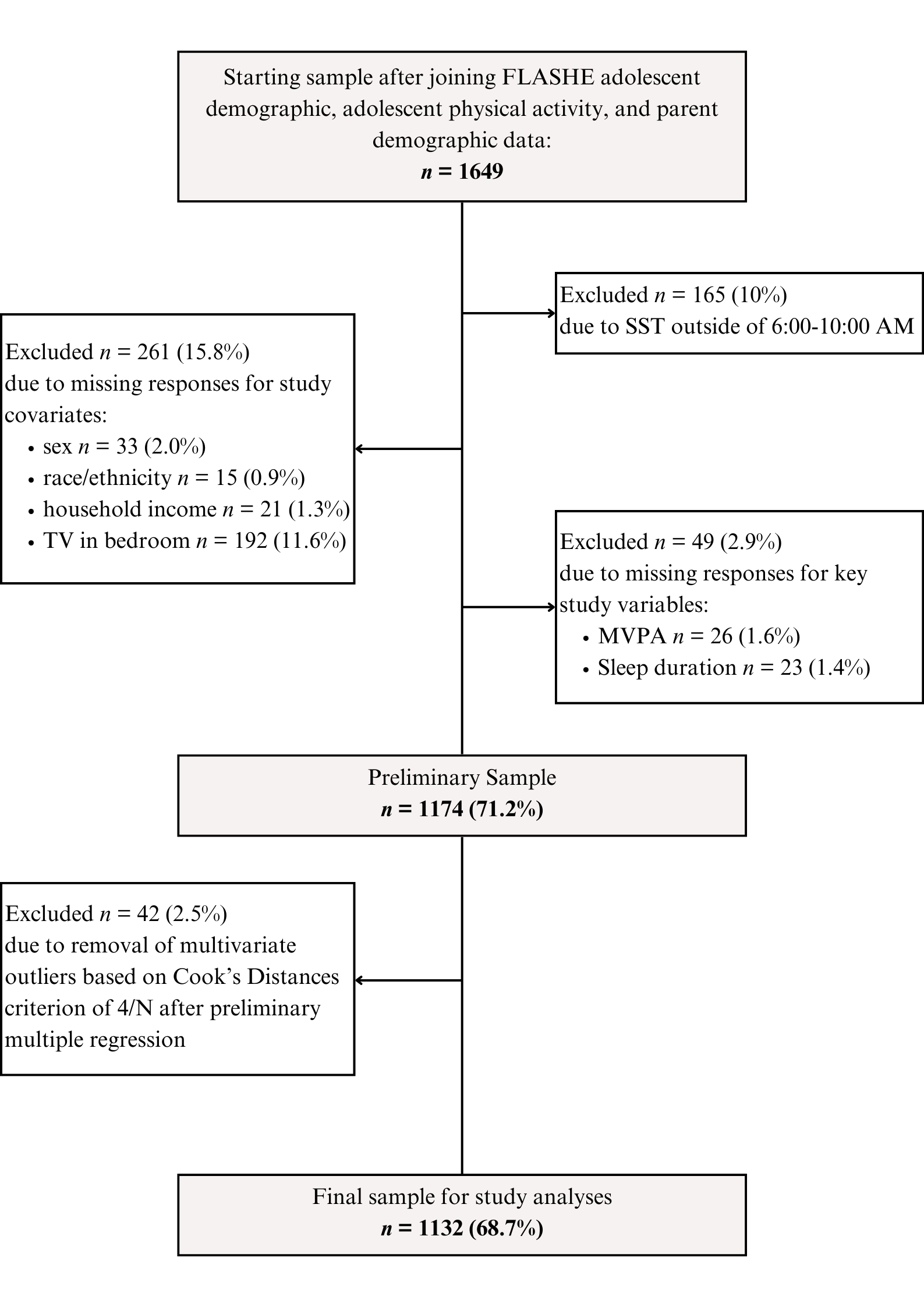

Supplement: Supplementary file 1 — Additional file 1. Inclusion-Exclusion Flow Chart for Final Sample. [file 44167_2024_50_MOESM1_ESM.png]
